# Relapsed cervicomediastinal lymph node carcinoma with an unknown primary site treated with TS-1 alone: a case report

**DOI:** 10.1186/1756-0500-6-558

**Published:** 2013-12-27

**Authors:** Toshiki Yajima, Ryoichi Onozato, Yoshinori Shitara, Akira Mogi, Shigebumi Tanaka, Hiroyuki Kuwano

**Affiliations:** 1 Department of General Surgical Science (Surgery I), Gunma University Graduate School of Medicine, 3-39-22 Showa-machi, 371-8511, Maebashi-shi, Gunma, Japan

**Keywords:** Mediastinal lymph node, Carcinoma with an unknown primary site, TS-1, Chemotherapy

## Abstract

**Background:**

Cervicomediastinal lymph node carcinoma with an unknown primary site is quite rare, and useful treatment of these diseases has not been established. We report here the case of a patient successfully treated with TS-1 alone after the relapse of cervicomediastinal lymph node carcinoma with an unknown primary site.

**Case presentation:**

A 62-year-old man was referred to our hospital because of cervicomediastinal lymph node swelling and high serum levels of carbohydrate antigen 19-9 and carcinoembryonic antigen. Fluorodeoxyglucose-positron emission tomography/computed tomography revealed an accumulation of fluorodeoxyglucose in the left supraclavicular lymph nodes, mediastinal lymph nodes, and the pelvic cavity. Colonoscopy revealed rectal cancer, which was diagnosed by biopsy as a tubular adenocarcinoma. Because metastases from rectal cancer to the cervicomediastinal lymph nodes are rare, the patient underwent thoracoscopic mediastinal lymphadenectomy. A biopsy specimen from the paraaortic lymph nodes demonstrated papillary adenocarcinoma that was pathologically different from the rectal cancer; therefore, a diagnosis of mediastinal carcinoma with an unknown primary site was established. The patient underwent low anterior resection of the rectum for the rectal cancer, and no abdominal lymph node metastasis (pMP, N0/stage I) was found. Although radiotherapy was performed for the cervicomediastinal lymph nodes, the mediastinal carcinoma relapsed after 6 months. Because the patient desired oral chemotherapy on an outpatient basis, TS-1 was administered at a dosage of 80 mg/day for 2 weeks, followed by a 1-week rest. TS-1 treatment resulted in a decrease in the size of the cervicomediastinal lymph nodes, and the serum tumor marker levels decreased to normal after the fourth course. The patient continued TS-1 treatment without adverse events and is currently alive without recurrence or identification of the primary site at the 32nd month after TS-1 treatment.

**Conclusion:**

This is the first reported case of relapsed cervicomediastinal lymph node carcinoma with an unknown primary site treated by TS-1 alone. TS-1 treatment for the carcinoma with an unknown primary site may be useful in patients who are not candidates for systemic platinum-based chemotherapy.

## Background

Metastatic carcinoma with an unknown primary site accounts for approximately 2%-10% of all solid malignancies; therefore, it is one of the 10 most frequent cancer diagnoses worldwide [1-4]. The patients present with metastatic disease for which a primary site cannot be detected at the time of diagnosis and have a poor prognosis (median survival of 6-9 months). Mediastinal lymph node (LN) carcinoma with an unknown primary site is rare, accounting for 1.5% of all carcinomas with an unknown primary site [5]. Several cases have been reported in which radical excision of the mediastinal LN carcinoma resulted in a better prognosis than for the primary lung cancer with mediastinal LN metastasis [6-8]. In contrast, some cases with multiple mediastinal LN carcinomas with an unknown primary site, which were not eligible for radical excision, had a poor prognosis [9]. Although a useful chemotherapy has not been established for multiple LN carcinomas with an unknown primary site, platinum-based doublet chemotherapy has often been employed [10,11]. TS-1 is an oral anticancer drug approved in Japan consisting of tegafur (a pro-drug of fluorouracil, 5-FU), gimeracil and oteracil potassium and has been mainly used for treatment of stomach cancer, non-small-cell lung cancer, colon and rectal cancer, head and neck cancer, inoperability or recurrence of breast cancer, and pancreatic cancer [12,13]. We report here the case of a patient with cervicomediastinal LN carcinoma with an unknown primary site that had relapsed after radiotherapy. The carcinoma was improved dramatically by TS-1 treatment alone.

## Case presentation

**Figure 1:**
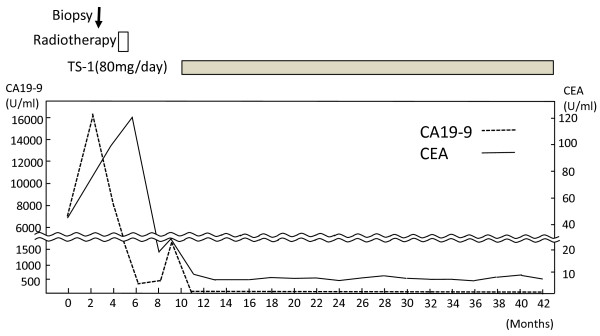
The levels of carcinoembryonic antigen (CEA) and carbohydrate antigen 19-9 (CA19-9) in the serum were investigated during the course of treatment.

**Figure 2:**
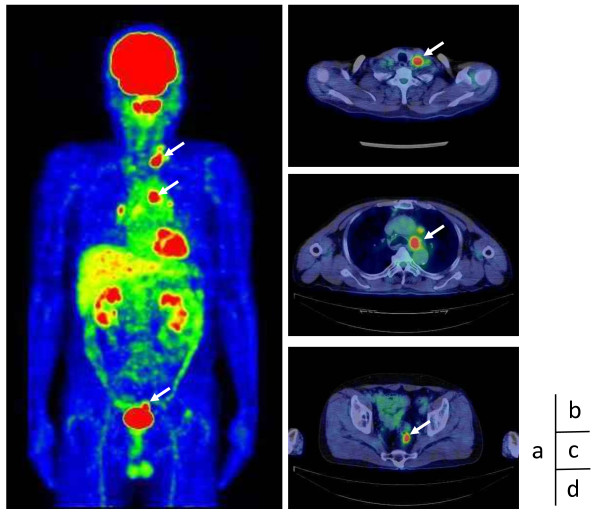
**Fluorodeoxyglucose (FDG)-positron emission tomography (PET)/computed tomography (CT) images.** Whole-body FDG-PET/CT demonstrating high uptake in the left supraclavicular region, mediastinal region, and rectal lesion (arrows) **(a)**, with maximum standardized uptake values of 10.44 **(b)**, 8.26 **(c)**, and 6.43 **(d)**, respectively.

**Figure 3:**
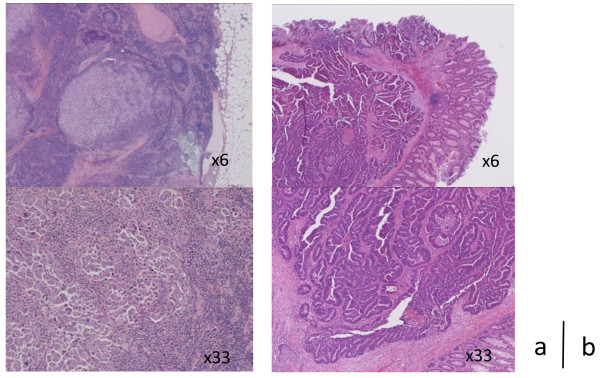
**Microscopic findings of resected mediastinal lymph nodes and the rectal tumor. (a)** A biopsy of the paraaortic LNs revealed papillary adenocarcinoma that was pathologically different from the rectal lesion (hematoxylin and eosin stain). **(b)** The pathological diagnosis of the tumor resected from the rectum was tubular adenocarcinoma.

A 62-year-old man was admitted to our hospital with hoarseness of voice in November 2008. Computed tomography (CT) revealed enlargement of LNs located at the left supraculavicular site, the level of Botallo’s ligament, and the left side of the ascending aorta, which had diameters of 18 mm, 18 mm, and 14 mm, respectively. The hoarseness was caused by left vocal cord palsy as a result of the compression of the left recurrent laryngeal nerve by enlarged Botallo’s LNs. The hematological, hepatic, and renal function tests were all within the normal range. The serum levels of carbohydrate antigen 19-9 (CA19-9) and carcinoembryonic antigen (CEA) were found to be dramatically elevated to 16149.0 U/ml and 72.7 U/ml, respectively (Figure 1). Fluorodeoxyglucose (FDG) - positron emission tomography (PET)/CT revealed an accumulation of FDG in the left supraclavicular LNs, mediastinal LNs, and the pelvic cavity, with maximum standardized uptake values of 8.26, 10.44, and 6.43, respectively (Figure 2). Colonoscopy revealed a rectal tumor 2 cm or less in diameter, which was diagnosed by biopsy as a tubular adenocarcinoma. The other primary site of cervicomediastinal LN carcinoma could not be determined on whole-body CT, upper gastrointestinal endoscopy, PET-CT, or urological examination. Although metastasis from infradiaphragmatic malignancies to cervicomediastinal LNs is rare, it was possible that the cervicomediastinal LN carcinoma had metastasized from rectal cancer. Biopsy specimens from the mediastinal LNs and left supraculavicular LNs were obtained by endobronchial ultrasound-guided transbronchial needle aspiration biopsy and echo-guided needle aspiration biopsy, respectively, but did not lead to a diagnosis. To identify the primary lesion for the mediastinal LN carcinoma, a thoracoscopic biopsy was performed in February 2009. The biopsy specimen from the paraaortic LNs demonstrated papillary adenocarcinoma that was pathologically different from rectal cancer (Figure 3). Additionally, immunohistochemistry (IHC) studies showed the biopsy specimen from the paraaortic LNs to be positive for thyroid transcription factor-1 (TTF-1) and cytokeratin 7 (CK7), and negative for cytokeratin 20 (CK20). These findings suggested the presence of metastatic adenocarcinoma of the LN originating from lung cancer. Therefore, the patient was diagnosed of mediastinal LN carcinoma from lung cancer with an unknown primary lesion. The patient underwent low anterior resection of the rectum for the rectal cancer, and no abdominal LN metastasis (pMP, N0/stage I) was found.

**Figure 4:**
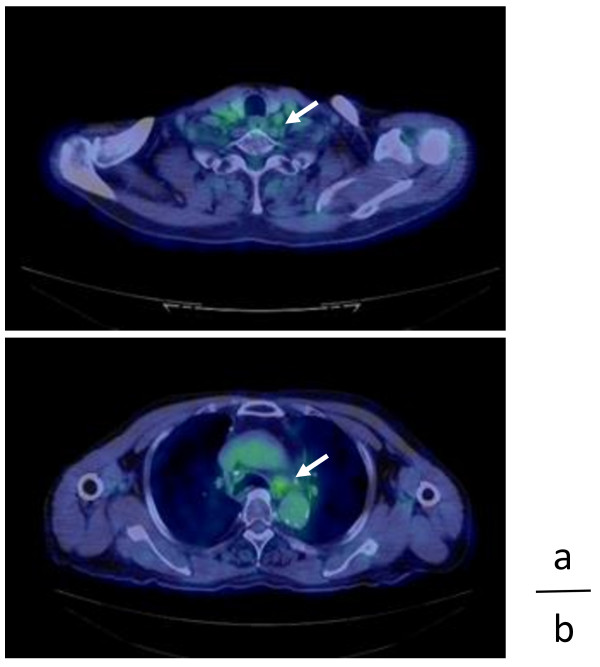
**Fluorodeoxyglucose (FDG)-positron emission tomography (PET)/computed tomography (CT) images at 12 months after TS-1 treatment.** FDG-PET/CT demonstrating in the FDG uptake of the left supraclavicular and Botallo’s ligament region, with maximum standardized uptake values of 2.32 (arrows) **(a)** and 2.26 (arrows) **(b)**, respectively.

Although we recommended concurrent radiotherapy with systemic platinum-based chemotherapy for the cervicomediastinal LN carcinoma, the patient refused; therefore, radiotherapy without chemotherapy was administrated with a total dose of 50 Gy (2 Gy x 25 fractions). The size of the LNs had decreased at 3 months after radiotherapy, and the serum levels of CA19-9 and CEA had decreased to 398.1 U/ml and 18.6 U/ml, respectively, in July 2009 (Figure 1). However, the serum levels of CA19-9 and CEA increased to 1599.2 U/ml and 23.3 U/ml, respectively, in August 2009 (Figure 1). CT was done again, and the result revealed regrowth of mediastinal LNs at 4 months after radiotherapy. Although we recommended systemic platinum-based chemotherapy for recurrence of the cervicomediastinal LN carcinoma, the patient desired oral chemotherapy on an outpatient basis. TS-1 was administered at a dosage of 80 mg/day for 2 weeks, followed by a 1-week rest, a treatment used for unresectable non-small cell lung cancer. The serum CA19-9 level decreased to the normal level, and the CEA level had also decreased to a normal or only slightly elevated value (5.9 U/ml) after the third course (Figure 1). No adverse effects were noted. After the end of the fourth course, CT revealed a marked decrease in the LN size and a complete response was achieved. The cervicomediastinal lesions showed a complete biologic response to TS-1 treatment after 12 courses as determined by PET/CT (Figure 4). The maximum standardized uptake value in the lesions in left supraclavicular region and Botallo’s ligament were 2.32 and 2.26, respectively. The serum levels of CA19-9 and CEA were maintained within the almost normal range during the 31 -months after TS-1 treatment (Figure 1). The patient continued treatment with oral TS-1 without adverse events and is currently alive without recurrence or identification of the primary site at the 32nd month after TS-1 treatment.

Mediastinal LN carcinoma with an unknown primary site is rare and comprises 1.5% of all cancers with unknown primary sites [1-3]. Seventy cases of mediastinal LN carcinoma with an unknown primary site have been reported in Japan [9]. There is a male preponderance, and the overall male to female ratio is 4.4:1. The median age at diagnosis is approximately 57.7 years (range 5-85 years). The histological type was adenocarcinoma in 41% of patients, followed by small cell carcinoma in 19%, large cell carcinoma in 17%, and squamous cell carcinoma in 16%. The mean survival time of patients with mediastinal LN carcinoma has been reported to be 28.9–30.7 months, and the prognosis of patients with mediastinal LN carcinoma with an unknown primary site is generally better than that of patients with primary lung cancer with mediastinal LN metastasis [6-9].

PET/CT is useful to determine the primary lesion for carcinoma with an unknown primary site [14]. In the present case, PET revealed an accumulation of FDG at the rectal lesion, which was diagnosed by biopsy as a tubular adenocarcinoma. Although it is possible that the cervicomediastinal LN carcinoma had metastasized from the rectal cancer, metastasis from infradiaphragmatic malignancies to cervicomediastinal LNs is rare and has never been reported to occur without abdominal LNs metastasis. A thoracoscopic biopsy of the mediastinal LNs was performed, and a biopsy specimen from the paraaortic LNs demonstrated papillary adenocarcinoma that was pathologically different from rectal cancer. Moereover, IHC studies is a useful addition to HE staining in locating the tumor site. In particular, a pattern of positive TTF-1 and CK7 expression and negative CK20 expression is indicative of primary lung cancer. These pathological results suggest that rectal cancer was not the origin of the LN metastasis; therefore, a diagnosis of mediastinal LN carcinoma with an unknown primary site was established.

A useful treatment for mediastinal LN carcinoma with an unknown primary site has not been established. Several surgical procedures including LN incisional biopsy, resection, dissection and lobectomy have been reported. Several case reports have described the complete excision of mediastinal LN carcinoma with a good prognosis [6-8]. Therefore, mediastinal carcinoma with an unknown primary site should be surgically excised if complete resection is possible. In the present case, complete excision of all of the metastatic LNs was impossible because supracervical LN lesions were prominent.

Several reports have demonstrated the effect of chemoradiotherapy on mediastinal LN carcinoma with an unknown primary site. Thoracoscopic biopsies of mediastinal LNs from patients with multiple LN lesions and subsequent concurrent chemoradiotherapy have indicated poor prognosis [9]. However, two patients with mediastinal LN carcinoma in multiple locations that were treated by chemoradiotherapy were reported to be alive at 24 and 33 months after the procedure [6]. Cervicomediastinal carcinoma with an unknown primary site was also successfully treated by chemotherapy with a platinum-based regimen followed by sequential radiotherapy [11]. These reports suggest that chemoradiotherapy may be useful for some cases with mediastinal LN carcinoma in multiple locations with an unknown primary site. Therefore, we recommended systemic platinum-based chemoradiotherapy to the patient, but the patient refused. We then applied radiotherapy to the LNs in the mediastinal and supraclavicular region. However, cervicomediastinal LNs in the present case showed regrowth at 4 months after radiotherapy, and the serum levels of CA19-9 and CEA increased again. There have been few reports of carcinoma with an unknown primary site in which TS-1 was effective [15]. Currently, TS-1 is administered for gastric, colorectal, lung, laryngeal, pancreatic, and biliary cancers in Japan. Because the patient desired oral chemotherapy on an outpatient basis, TS-1 was administered after the cervicomediastinal LN carcinoma had relapsed after radiotherapy. After the initiation of TS-1 treatment, PET/CT revealed a marked decrease in LN size and in the FDG uptake of the cervicomediastinal lesions; a complete biological response was achieved (Figure 4). The serum levels of CA19-9 and CEA were maintained within almost the normal range during the 31-months after TS-1 treatment. Therefore, this regimen was considered to show clinical efficacy. At the 32nd month after TS-1 treatment, there was no exacerbation, and the quality of life has been maintained. TS-1 treatment for mediastinal LN carcinoma with an unknown primary site may be recommended as therapeutic strategy. To our knowledge, this is the first case report in the English-language medical literature to describe a patient successfully treated with TS-1 alone after the relapse of cervicomediastinal LN carcinoma with an unknown primary site.

## Conclusion

TS-1 treatment for the carcinoma with an unknown primary site may be useful in patients who are not candidates for systemic platinum-based chemotherapy, because TTF1+ carcinoma patients are treated like non-small cell lung cancer patients.

## Consent

Written informed consent was obtained from the patient for publication of this case report and any accompanying images. A copy of the written consent is available for review by the Editor-in-Chief of this journal.

## Competing interests

The authors declare that they have no competing interests.

## Authors’ contributions

TY was a major contributor in writing the manuscript and giving the diagnosis. TY and YS performed the operation. RO interpreted the patient data. ST was responsible for the overall treatment of the patient and edited the manuscript. AM analyzed data and edited the manuscript. KH was responsible for all aspects of management as chief of the Department of General Surgical Science (Surgery I) Gunma University Graduate School of Medicine. All authors read and approved the final manuscript.

## References

[B1] Pavlidis N Pentheroudakis G Cancer of unknown primary site Lancet 2012 6 1428 1435 10.1016/S0140-6736(11)61178-1 22414598

[B2] Holmes FF Fouts TL Metastatic cancer of unknown primary site Cancer 1970 6 816 820 10.1002/1097-0142(197010)26:4<816::AID-CNCR2820260413>3.0.CO;2-R 5506606

[B3] Didolkar MS Fanous N Elias EG Moore RH Metastatic carcinomas from occult primary tumors. A study of 254 patients Ann Surg 1977 6 625 630 10.1097/00000658-197711000-00014 921356 PMC1396304

[B4] Pavlidis N Fizazi K Carcinoma of unknown primary (CUP) Crit Rev Oncol Hematol 2009 6 271 278 10.1016/j.critrevonc.2008.09.005 18977667

[B5] Riquet M Badoual C Le Pimped BF Dujon A Danel C Metastatic thoracic lymph node carcinoma with unknown primary site Ann Thorac Surg 2003 6 244 249 10.1016/S0003-4975(02)04119-X 12537223

[B6] Miwa K Fujioka S Adachi Y Haruki T Taniguchi Y Nakamura H Mediastinal lymph node carcinoma of an unknown primary site: clinicopathological examination Gen Thorac Cardiovasc Surg 2009 6 239 243 10.1007/s11748-008-0361-5 19440819

[B7] Nakano T Endo S Endo T Hasegawa T Nakayama M Sugiyama Y Hironaka M Multimodal treatment for multistation mediastinal lymph node adenocarcinoma: a case report Ann Thorac Cardiovasc Surg 2012 6 136 139 10.5761/atcs.cr.11.01668 22001212

[B8] Tomita M Matsuzaki Y Shimizu T Hara M Ayabe T Enomoto Y Onitsuka T Squamous cell carcinoma of the hilar lymph node with unknown primary tumor: a case report Ann Thorac Cardiovasc Surg 2008 6 242 245 18818574

[B9] Miyoshi K Okumura N Kokado Y Matsuoka T Kameyama K Metastatic thoracic lymph node carcinoma of unknown origin Jpn J Lung Cancer 2007 6 245 250 (in Japanese) 10.2482/haigan.47.245

[B10] Lee J Hahn S Kim DW Kim J Kang SN Rha SY Lee KB Kang JH Park BJ Evaluation of survival benefits by platinums and taxanes for an unfavourable subset of carcinoma of unknown primary: a systematic review and meta-analysis Br J Cancer 2013 6 39 48 10.1038/bjc.2012.516 23175147 PMC3553519

[B11] Shiota Y Imai S Sasaki N Tahara K Noma B Horita N Taniguchi A Ono T A case of mediastinal lymph node carcinoma of unknown primary site treated with docetaxel and cisplatin with concurrent thoracic radiation therapy Acta Med Okayama 2011 6 407 411 22189482 10.18926/AMO/47267

[B12] Satoh T Sakata Y S-1 for the treatment of gastrointestinal cancer Expert Opin Pharmacother 2012 6 1943 1959 10.1517/14656566.2012.709234 22860709

[B13] Okamoto I Fukuoka M S-1: a new oral fluoropyrimidine in the treatment of patients with advanced non-small-cell lung cancer Clin Lung Cancer 2009 6 290 294 10.3816/CLC.2009.n.040 19632949

[B14] Moller AK Loft A Berthelsen AK Damgaard Pedersen K Graff J Christen CB Perell K Petersen BL Daugaard G 18F-FDG PET/CT as a diagnostic tool in patients with extracervical carcinoma of unknown primary site: a literature review Oncologist 2011 6 445 451 10.1634/theoncologist.2010-0189 21427201 PMC3228126

[B15] Kawasaki K Kamigaki T Takase S Maki K Tamura T Shirasaka D Shinoda H Nakamura T Kuroda D Kuroda Y A case of unknown primary cancer responding to TS-1 Gan To Kagaku Ryoho 2006 6 1125 1128 (in Japanese) 16912532

